# Supplementary material for: Spatial clustering of notified tuberculosis in Ethiopia: A nationwide study
Source: PLoS One. 2019 Aug 9;14(8):e0221027. doi: 10.1371/journal.pone.0221027 (PMC6688824; doi:10.1371/journal.pone.0221027)
Supplement: S1 Table — (DOCX) [file pone.0221027.s001.docx]

**S1 Table:** Summary of independent variables, sources of data and definition of variables

| **Independent variables** | **Data sources** | **Definition** |
| --- | --- | --- |
| Socio-economic factors | | |
| Low wealth index | EDHS 2016 | Total number of people with low wealth index (poorer and poorest) divided by the total number of people participated in the survey. |
| Population density | Ethiopia Atlas of Population Density | Number of people per square kilometre |
| Dependency ratio | Ethiopia Atlas of Population Density | Number of children (aged under 15 years) and elderly (aged 65+) dividing by the working-age population (aged 15-64 years) |
| Average number of persons per room | Ethiopia Atlas of Population Density | Average number of people living in a room |
| Unemployed population | Ethiopia Atlas of Population Density | Percentage of people in the labour force who were unemployed |
| Adult literacy rate | Ethiopia Atlas of Population Density | Percentage of population aged 15 years and above who had attended higher than secondary school or who can read and write a short simple statement |
| Behavioural factors | | |
| Chat chewing | EDHS 2016 | Total number of people chewing chat in the last one month prior to the survey divided by the total number of people participating in the survey |
| Alcohol drinking | EDHS 2016 | Total number of people drinking alcohol in the month prior to the survey divided by the total number of people participating in the survey |
| Health care access and knowledge and attitude regarding TB | | |
| Health care access problem | EDHS 2016 | Difficulty of getting advice or treatment due to lack of money, or distance to a health facility |
| Good knowledge toward TB | EDHS 2011 | Number of people with good knowledge towards TB divided by the total number of people participating in the survey. |
| Good attitude towards TB | EDHS 2011 | Number of people with good attitude towards TB divided by the total number of people participating in the survey |
| Climatic and environmental factors | | |
| Enhanced vegetation index | EDHS Spatial Analysis data | The average enhanced vegetation index which is calculated by measuring the density of green leaves in the near-infrared and visible bands. |
| Rainfall | EDHS Spatial Analysis data | Annual mean rainfall (mm) |
| Aridity | EDHS Spatial Analysis data | The average aridity index calculated by dividing the actual evapotranspiration by the potential evapotranspiration. |
| Mean temperature | EDHS Spatial Analysis data | Annual mean environmental air temperature (°C). |
