# Supplementary material for: Spatial clustering of notified tuberculosis in Ethiopia: A nationwide study
Source: PLoS One. 2019 Aug 9;14(8):e0221027. doi: 10.1371/journal.pone.0221027 (PMC6688824; doi:10.1371/journal.pone.0221027)
Supplement: S2 Table — (DOCX) [file pone.0221027.s002.docx]

**S2 Table:** The incidence rate of bacteriologically confirmed and all forms of tuberculosis in Ethiopia at zone level and region level, between June 2016 and June 2017.

|  |  | **All forms of TB incidence rate per 100000** | **Bacteriologically confirmed TB Incidence rate per 100 000** |  |
| --- | --- | --- | --- | --- |
|  | **Ethiopia** | 112.7 | 41.2 |  |
|  | **Addis Ababa** | 262.1 | 70.7 |  |
|  | **Afar** | 156.4 | 51.8 |  |
|  | Zone 05 | 57.4 | 31.8 |  |
|  | Zone 03 | 114.9 | 49.6 |  |
|  | Zone 04 | 120.3 | 41.2 |  |
|  | Zone 01 | 168.1 | 73.6 |  |
|  | Zone 02 | 211.7 | 48.4 |  |
|  | **Amhara** | 108.3 | 25.7 |  |
|  | North Shewa | 83.1 | 30.5 |  |
|  | North Wello | 85.1 | 18.9 |  |
|  | Waghemira | 96.2 | 10.1 |  |
|  | East Gojjam | 98.2 | 25.1 |  |
|  | South Wello | 109.4 | 28.4 |  |
|  | South Gondar | 111.8 | 19.7 |  |
|  | West Gojjam | 113 | 23.9 |  |
|  | North Gondar | 113.1 | 27.7 |  |
|  | Argoba Special woreda | 115 | 89.7 |  |
|  | Awi | 118.7 | 22.9 |  |
|  | Oromiya | 156.1 | 39.1 |  |
|  | Bahir Dar Special | 221.6 | 48.9 |  |
|  | **Beneshangul Gumu** | 61.9 | 21.1 |  |
|  | Maokomo Special | 28.1 | 11.1 |  |
|  | Kamashi | 31.9 | 13.9 |  |
|  | Assosa | 49 | 24.6 |  |
|  | Pawe Special | 81.4 | 19 |  |
|  | Metekel | 108.7 | 26.4 |  |
|  | **Dire Dawa** | 348.5 | 85.1 |  |
|  | **Gambela** | 152.7 | 73.9 |  |
|  | Nuwer | 41.6 | 20.8 |  |
|  | Etang Spe. | 82.7 | 47.6 |  |
|  | Mejenger | 137.5 | 69.5 |  |
|  | Agnewak | 321.1 | 150.1 |  |
|  | **Harer** | 206.7 | 61.9 |  |
|  | **Oromiya** | 115.2 | 44.6 |  |
|  | Illu Aba Bora | 76.8 | 22.1 |  |
|  | West Hararge | 85.1 | 36.9 |  |
|  | West Wellega | 89.6 | 33.6 |  |
|  | Jimma | 91.2 | 44.6 |  |
|  | Bale | 92.8 | 36.3 |  |
|  | West Shewa | 95.3 | 32.3 |  |
|  | Horo Gudru Wellega | 103.3 | 24.7 |  |
|  | North Shewa | 106.9 | 45.4 |  |
|  | South West Shewa | 110.4 | 37.9 |  |
|  | West Arsi | 115.7 | 45.8 |  |
|  | East Hararge | 120 | 42.3 |  |
|  | Borena | 121.4 | 78.7 |  |
|  | East Wellega | 125.3 | 23.9 |  |
|  | Kelem Wellega | 127.9 | 40.7 |  |
|  | Arsi | 132 | 50.3 |  |
|  | Guji | 153 | 84.5 |  |
|  | East Shewa | 166.1 | 53.9 |  |
|  | Burayu Special | 242.3 | 69.2 |  |
|  | Jimma Spe Town | 346.3 | 129.8 |  |
|  | Adama Special | 354.3 | 98.1 |  |
|  | **SNNP** | 101.4 | 56.6 |  |
|  | Konso Special | 10.8 | 5.6 |  |
|  | Basketo | 15.8 | 12.4 |  |
|  | Dawro | 34.7 | 18.6 |  |
|  | Yem Special Wereda | 38.9 | 17.5 |  |
|  | Konta | 44.8 | 27.6 |  |
|  | South Omo | 66.8 | 36.4 |  |
|  | Keffa | 67.2 | 39.2 |  |
|  | Awassa Town | 68 | 45.2 |  |
|  | Gamo Gofa | 71.2 | 41.6 |  |
|  | Amaro Special | 81.8 | 53.1 |  |
|  | Siliti | 84.6 | 33.8 |  |
|  | Kembata Tembaro | 98.1 | 52.8 |  |
|  | Alaba | 100.7 | 47.8 |  |
|  | Wolayita | 100.7 | 61.8 |  |
|  | Sidama | 119.5 | 75.2 |  |
|  | Derashe Lyiu Wereda | 119.9 | 56.9 |  |
|  | Hadiya | 124.5 | 50.3 |  |
|  | Bench Maji | 132.2 | 79.6 |  |
|  | Gurage | 141.6 | 52.3 |  |
|  | Sheka | 146.5 | 73.4 |  |
|  | Gedeo | 161.7 | 119.8 |  |
|  | Burji Special | 258.1 | 110.6 |  |
|  | **Somali** | 51.1 | 18.9 |  |
|  | Liben | 8.9 | 4.1 |  |
|  | Korahe | 13.4 | 3.5 |  |
|  | Warder | 18.8 | 8.1 |  |
|  | Gode | 38.7 | 23.5 |  |
|  | Afder | 40.7 | 30.1 |  |
|  | Shinile | 41.4 | 15.2 |  |
|  | Degehabur | 52.2 | 27.5 |  |
|  | Jijiga | 118.3 | 21.1 |  |
|  | Fik | NA | NA |  |
|  | Shinile | NA | NA |  |
|  | **Tigray** | 100.8 | 22.4 |  |
|  | South East | 75.4 | 14.5 |  |
|  | South Tigray | 81.1 | 13.1 |  |
|  | Central Tigray | 82.1 | 20 |  |
|  | North Western Tigray | 93.2 | 32.1 |  |
|  | Eastern Tigray | 94.7 | 19.7 |  |
|  | Mekele Especial Zone | 175.7 | 27.3 |  |
|  | Western Tigray | 191.3 | 42.3 |  |
|  | | | | |
