# Supplementary material for: Spatial clustering of notified tuberculosis in Ethiopia: A nationwide study
Source: PLoS One. 2019 Aug 9;14(8):e0221027. doi: 10.1371/journal.pone.0221027 (PMC6688824; doi:10.1371/journal.pone.0221027)
Supplement: Attachment — Submitted filename: Response to Reviewers.docx [file pone.0221027.s003.docx]

**Manuscript Number:** PONE-D-19-17592

**Article Title:** Spatial clustering of notified tuberculosis in Ethiopia: a nationwide study

**Authors:** Kefyalew Addis Alene, Archie CA Clements

**Journal:** PLOS ONE

**Response to reviewers' comments:**

**Reviewer #1:** the article is technically sound and statistical analysis been performed appropriately and rigorously

***Response:*** We greatly appreciate the reviewer’s efforts to carefully review the paper.

**Reviewer #2:** Introduction: remove the word "end" in the phrase "short course (DOTS) strategy since 1994 and [5, 6]"

***Response:*** This is now corrected.

**Discussion:** change the word "visualisation" with "visualization"

**Response:** This is also now corrected.
